# Effect of single parenteral administration of marbofloxacin on bacterial load and selection of resistant *Enterobacteriaceae* in the fecal microbiota of healthy pigs

**DOI:** 10.1186/s12917-024-04329-9

**Published:** 2024-10-29

**Authors:** Micaela Miyauchi, Farid EL Garch, William Thériault, Bruno G. Leclerc, Edith Lépine, Henry Giboin, Mohamed Rhouma

**Affiliations:** 1 Department of Pathology and Microbiology, Faculty of Veterinary Medicine, Université de Montréal, Saint-Hyacinthe, QC J2S 2M2 Canada; 2 Groupe de Recherche et d’Enseignement en Salubrité Alimentaire (GRESA), Faculty of Veterinary Medicine, Université de Montréal, Saint-Hyacinthe, QC J2S 2M2 Canada; 3 Swine and Poultry Infectious Diseases Research Center, Faculty of Veterinary Medicine, Université de Montréal, Saint-Hyacinthe, QC J2S 2M2 Canada; 4 Scientific Division, Vetoquinol S.A., Lure, France; 5 Vetoquinol N.-A. Inc., Scientific Affairs, Lavaltrie, Québec Canada

**Keywords:** Marbofloxacin, *Escherichia coli*, Antimicrobial resistance, Pigs

## Abstract

**Background:**

Antimicrobial resistance (AMR) is a global concern impacting both humans, animals and their environment. The use of oral antimicrobials in livestock, particularly in pigs, has been identified as a driver in the selection of AMR bacteria. The aim of the present study was to evaluate the effects of a single intramuscular (IM) dose of marbofloxacin (8 mg/kg) on *Enterobacteriaceae* and *E. coli* populations, as well as on fluoroquinolone resistance within the fecal microbiota of pigs. Twenty healthy pigs, 60-days old, were divided into two groups: a treated group (*n* = 13) and a control group (*n* = 7) and were monitored over a 28-day experimental period. Fecal samples were collected from all animals for the isolation of *E. coli* and *Salmonella* strains. The minimum inhibitory concentration (MIC) of marbofloxacin for the isolates recovered on MacConkey agar supplemented with 1 or 4 µg/mL of marbofloxacin and for some generic *E. coli* isolates (recovered from MacConkey agar not supplemented with marbofloxacin) was determined using the broth microdilution method. Genomic DNA was extracted from the confirmed bacterial strains and sequenced using the Sanger method to identify mutations in the quinolone resistance determining regions (QRDRs) of the *gyrA* and *parC* genes.

**Results:**

The single IM administration of marbofloxacin resulted in a significant decrease in *Enterobacteriaceae* and *E. coli* fecal populations from days 1 to 3 post- treatment. No *Salmonella* isolates were detected in either group, and no marbofloxacin-resistant *E. coli* isolates were identified. The MIC of the selected generic *E. coli* strains (*n* = 100) showed an increase to up to 0.5 µg/mL between days 1 and 3 post-treatment but remained below the clinical breakpoint of marbofloxacin resistance (4 µg/mL). Sequencing of these isolates revealed no mutations in *gyrA* and *parC* genes.

**Conclusions:**

The present study showed that this dosing regimen of marbofloxacin significantly decreases the fecal shedding of *Enterobacteriaceae* and *E. coli* populations in pigs, while limiting the selection of marbofloxacin-resistant *E. coli* isolates. These findings warrant validation in sick pigs to support the selective use of this antibiotic solely in cases of clinical disease, thereby minimizing the reliance on conventional (metaphylactic) group treatments in pigs.

## Background

Antimicrobial resistance (AMR) is a global concern affecting human, animal and environmental health [1]. In 2021, the World Health Organization (WHO) identified AMR as one of the top 10 major threats to the global healthcare system [2], eventually resulting in the publishing, in June 2023, of its first global research agenda to tackle AMR, outlining 40 research priorities to be addressed by 2030 [3, 4]. Around the same time, recognizing the interconnected cross-sectoral nature of AMR, the WHO, the Food and Agriculture Organization (FAO), the World Organisation for Animal Health (WOAH), and the United Nations Environment Programme (UNEP) – collectively known as the Quadripartite –published, in July 2023, the One Health Priority Research Agenda for AMR report [5]. In addition to causing death and impairing therapeutic success, AMR entails considerable economic costs [6, 7]. In fact, it has been estimated that AMR could result in additional healthcare costs of US$ 1 trillion by 2050, and gross domestic product (GDP) losses of US$ 1 trillion to US$ 3.4 trillion per year by 2030 [8]. The misuse of antimicrobials in human healthcare, in animals and plants is now recognized as the main driver of AMR [9, 10]. However, ongoing research aims to elucidate how farm animals contribute to the spread of AMR in humans. Studies suggest that antimicrobial misuse in animals—including unnecessary use, overuse of medically important antimicrobials, subinhibitory dosage, and inadequate treatment duration—plays a significant role in selecting for AMR bacteria and their resistance determinants [11].

Therefore, to preserve the longevity and efficacy of antimicrobials in animal productions, several actions have been implemented to decrease antimicrobial use (AMU) and minimize the selection of AMR bacteria, all while adopting a One Health approach [12, 13]. In addition, some regulatory agencies now require that the approval process for new veterinary antimicrobials includes assessing their impact on bacteria of human health concern [14]. In pigs, most antimicrobials are used orally, and are generally employed for metaphylaxis purposes to prevent infections in healthy pigs while treating sick pigs housed in the same pens [15]. Although oral antimicrobials are a highly practical tool for controlling bacterial infections in pigs, this route of administration can be associated with a disturbance in the equilibrium of the intestinal ecosystem, the selection of resistant intestinal bacteria (including important zoonotic microorganisms), as well as the interaction of antimicrobials with certain feed constituents such as cellulose [16–18]. Moreover, several studies have highlighted the difficulty of ensuring homogeneous exposure of pigs to orally administered antimicrobials, along with concerns about the stability of these molecules in medicated feed and drinking water on pig farms [15, 19]. Among ways of overcoming such disadvantages, numerous antimicrobial products have been approved in pigs for the parenteral treatment of bacterial infections, including those with a concentration-dependent mode of action, such as fluroquinolones. These molecules are of particular interest, as their administration requires only a single dose, thus minimizing the need for repeated handling of pigs and reducing unnecessary stress in treated animals [20, 21].

Within this framework, marbofloxacin, a broad-spectrum synthetic third-generation fluoroquinolone antibiotic, is approved in the European Union (EU) for use in pigs for the parenteral treatment of respiratory infections and Mastitis Metritis Agalactiae syndrome in sows [22], as well as for the treatment of intestinal infections caused by *E. coli* in weaned piglets, at a dose of 8 mg/kg bodyweight (bw) (1 mL/20 kg (bw)) through a single intramuscular (IM) administration [23]. Additionally, marbofloxacin is approved for dogs and cats in many countries including the USA and Canada [24], and for the individual treatment of bovine respiratory disease (BRD) associated with *Mannheimia haemolytica* or *Pasteurella multocida* in infected cattle in Canada [25]. Some studies also reported an extra-label use of marbofloxacin in other farm animals (e.g., sheep for the treatment of endotoxemia, goat for the treatment of mastitis and horses to treat certain infections caused by susceptible bacteria) [26]. Marbofloxacin is a bactericidal molecule active against a wide range of Gram-negative and Gram-positive bacteria, including *Mycoplasma*, and acts by inhibiting bacterial type II topoisomerases, DNA gyrase and topoisomerase IV [27, 28]. As with all fluoroquinolones, the most common mechanism of resistance to this antimicrobial is most often associated with specific mutations in the essential bacterial enzymes DNA gyrase (*gyrA* and *gyrB*) and/or DNA topoisomerase IV (*parC* and *parE*) [28]. These mutations involve amino acid substitutions in subregions of bacterial type II topoisomerases, known as the “quinolone-resistance—determining region” (QRDR) [29]. The clinical breakpoint of marbofloxacin resistance, used to categorize aerobic pathogenic Gram-positive or Gram-negative bacteria as resistant (R) in farm animals and pets, is 4 µg/mL [26, 27].

Some studies have been previously carried out in pigs to assess the effect of a single IM administration of marbofloxacin (8 mg/kg (bw)) in the treatment of acute porcine pleuropneumonia associated with *Actinobacillus pleuropneumoniae* [20], as well as in the treatment of some respiratory disease in pigs [30]. However, to the best of our knowledge, no scientific study has evaluated until now the effect of this therapeutic regimen on fecal *Enterobacteriaceae* concentrations in fattening pigs, or its impact on the evolution of resistance within this intestinal bacterial population. Therefore, the aim of the current study was to evaluate the effects of a single IM therapeutic dose of marbofloxacin in pigs on the fecal shedding of *Enterobacteriaceae* and *E. coli* populations, as well as the development of fluoroquinolone resistance in *Enterobacteriaceae* bacteria such as *E. coli* and *Salmonella* derived from the fecal microbiota of the treated healthy fattening pigs.

## Materials and methods

### Ethical approval

The experimental protocol (21-Rech-2146) of the present study was reviewed and approved by the Ethics Committee on Animal Use of the Faculty of Veterinary Medicine (FVM) of the Université de Montréal and performed in accordance with the guidelines of the Canadian Council on Animal Care (CCAC). It is noteworthy that marbofloxacin was not approved for use in swine in Canada during the project period (2021–2022). This drug was therefore used in the current study following the obtention of an experimental studies certificate (ESC) from the Veterinary Drugs Directorate (VDD) of Health Canada.

### Animals, housing, and experimental design

**Fig. 1:**
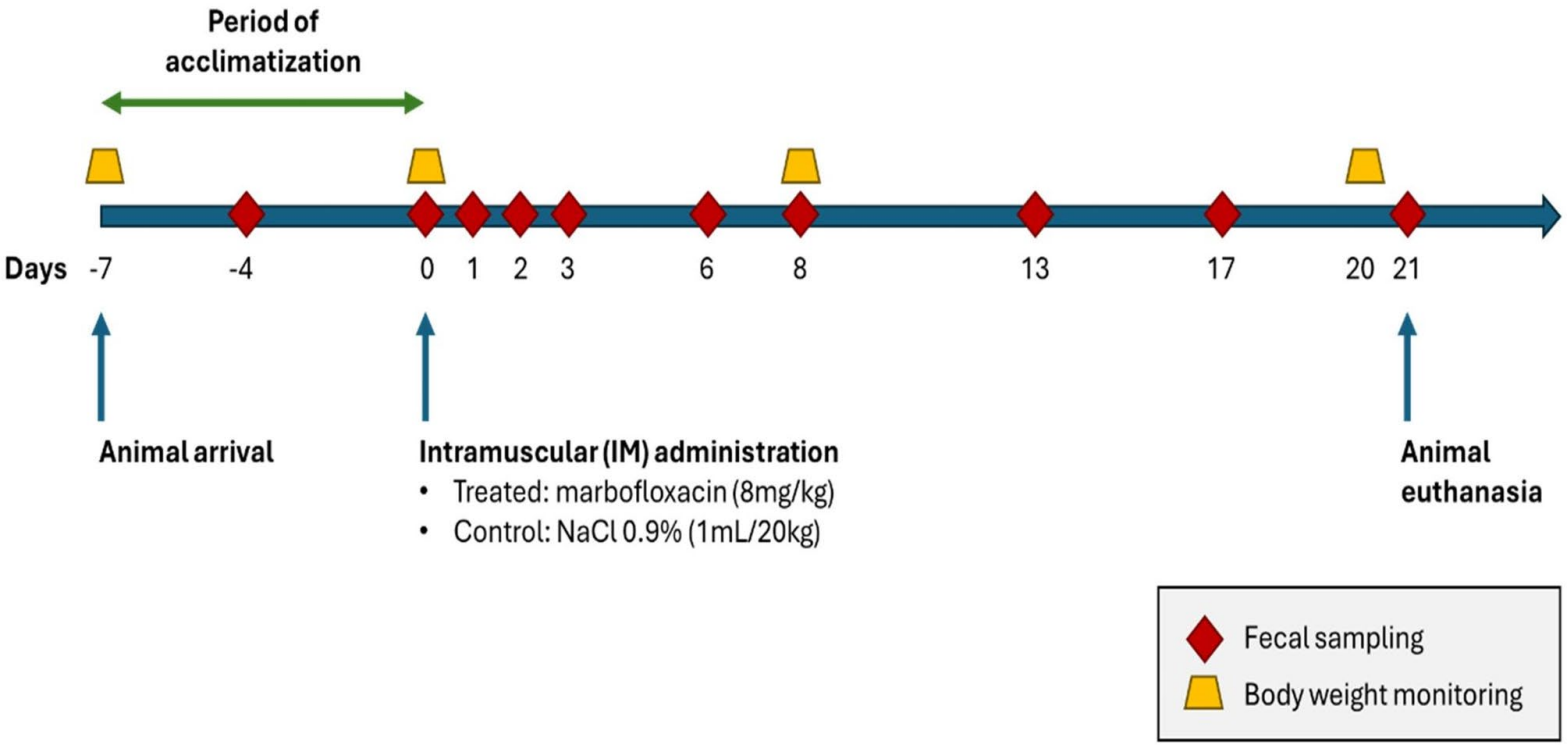
The experimental design. Treatment with marbofloxacin (8 mg/kg) or NaCl 0.9% (1 mL/20 kg) was administered intramuscularly (IM) on D0 of the experiment

A total of 20 pigs (60 days old - end of the nursery period (32.98 ± 1.44 kg)) were purchased from an antibiotic-free farm located in the Montérégie region (Québec, Canada) and were used in this study. The choice of this antibiotic-free farm was motivated by the need to avoid any interaction between marbofloxacin and other antimicrobials likely to be administered to pigs during this critical period in pig production on conventional farms. Animals were housed at a biosecurity level 2 agro-environmental platform for farm animals of the FVM. Pigs were fed a standard non-medicated diet free of feed additives (e.g., zinc oxide) and had unlimited access to feed and water during the 4-week study. Two groups of animals were randomly assigned: (1) treated group (*n* = 13) and (2) control group (*n* = 7). The two groups were placed in two different rooms: room 1655-1 for the control group, and room 1655-3 for the treated group. No material (e.g., boots, coveralls, gloves) was transferred between the two rooms. To avoid cross-contamination between groups, room 1655-1 was visited first, followed by room 1655-3, and this order was respected throughout the study. Each animal was identified by an ear tag to facilitate its monitoring and the subsequent presentation of results at individual pig level. Ear tag numbers 72 to 78 were used to identify the control group, while ear tag numbers 79 to 91 were used to identify the treated group. After 1-week of acclimatization, pigs in the treated group received a single IM injection of 8 mg/kg of marbofloxacin (1 mL/20 kg) (160 mg/mL, Forcyl^®^, Vetoquinol N.-A. Inc.) and pigs in the control group received a single IM injection of a commercial physiological solution (NaCl 0.9%) (1 mL/20 kg) (Fig. 1). The IM administrations were carried out on the pigs’ necks. Pigs in both groups were weighed before the IM administrations to adjust doses for each pig.

Throughout the study, the pigs’ general conditions, rectal temperatures, and body weights were recorded. In addition, injection sites in both groups were monitored daily for adverse changes (e.g., tumefaction, nodule, abscess, induration, lesions). Fecal samples (equivalent to 40 g) were collected directly from the rectum of each animal after stimulation of its defecation reflex with a thermometer. Fecal samples were collected before the single IM administration of marbofloxacin (at D-4 and D0) and at days D1, D2, D3, D6, D8, D13, D17 and D21 after the IM administration of marbofloxacin (Fig. 1). Changes in body weight were recorded for each pig throughout the experiment at D-7, D0, D8 and D20 (Fig. 1). All animals were euthanized at the end of the experiment (D21). In fact, each pig was initially sedated by a combined IM administration of ketamine (20 mg/kg) and xylazine (2 mg/kg). Euthanasia was then performed in an antechamber where sedated animals were euthanized by an overdose of anesthetic through intracardiac administration of phenobarbital (110 mg/kg).

### Bacterial isolation and identification

Fecal samples were used for the enumeration of *Enterobacteriaceae* using violet-red bile glucose (VRBG) agar (Biokar diagnostic, Allonne, France) and the coliform bacteria using MacConkey agar (Biokar diagnostic, Allonne, France), as well as for the detection of *Salmonella* using both brilliant green sulfa (BGS) agar plates (Fisher scientific, Ottawa, ON, Canada) and xylose lysine desoxycholate (XLD) agar plates (Innovation scientific, Saint-Eustache, QC, Canada), as previously described [31]. For the enumeration of *Enterobacteriaceae* and the generic *E. coli* population, each fecal sample was homogenised 1/10 (weight/volume) in buffered peptone water and filtered using a Whirl-pak sterile sample bag. The filtrate (100 µL) was streaked on VRBG agar and MacConkey plates and incubated under aerobic conditions at 37 °C for 20-24 h. The total number of colonies growing on both sets of plates was manually counted. It should be noted that only colonies with a characteristic morphology of *E. coli* (lactose-positive isolates (pink)), were counted on MacConkey agar. When detected on MacConkey agar plates, 3 to 4 colonies were recovered and purified using blood agar plates (Fisher Scientific, Ottawa, ON, Canada), then identified by Matrix-Assisted Laser Desorption Ionization-Time-of-Flight Mass Spectrometry (MALDI-TOF MS) analysis as previously described [32].

Isolation of *Salmonella* from each fecal sample was carried out according to the protocol previously described in our laboratory by Boubendir et al. [31]. Briefly, 10 g of each fecal sample were pre-enriched in a 1:10 ratio of buffered peptone water and incubated at 37 °C for 24 h. The enriched culture was inoculated on the modified semisolid rappaport-vassiliadis (MSRV) agar plates (Innovation diagnostics, Saint-Eustache, QC, Canada) and incubated at 41.5 °C for 24 h under aerobic conditions. Following incubation, MSRV plates were verified for any evidence of bacterial growth, resulting in the appearance of a white migratory zone. Plates were re-evaluated after an additional 24 h of incubation if no migration zone was observed after the first incubation. In parallel, 1 mL of the enriched culture was diluted into 9 mL of sterile tetrathionate brilliant green (TBG) bile broth (Fisher Scientific, Ottawa, ON, Canada) and incubated at 37 °C for 24 h under aerobic conditions. TBG broth and positive MSRV were then inoculated on BGS agar (Fisher scientific, Ottawa, ON, Canada) and xylose lysine deoxycholate (XLD) (Innovation scientific, Saint-Eustache, QC, Canada), and latter incubated at 37 °C for 24 h under aerobic conditions. BGS agars were incubated for a supplemental 24 h when no typical colonies were observed. When presumptive *Salmonella* isolates were available on BGS agar and/or XLD agar plates, 3 colonies per selective agar plate were recovered. Triple sugar iron agar slants (Innovation scientific, Ottawa, ON, Canada) and urea agar slants (Innovation scientific, Ottawa, ON, Canada) were used as biochemical tests to confirm the identity of these isolates. Presumed *Salmonella* isolates were plated on sheep blood agar plates (Fisher Scientific, Ottawa, ON, Canada) and identified by MALDI-TOF MS analysis.

### Antimicrobial susceptibility testing

#### Phenotypic susceptibility determination

In parallel with the bacterial isolation described in the above section, MacConkey and BGS agars plates containing marbofloxacin at 1 or 4 µg/mL were used to enumerate intermediate and resistant *E. coli* and *Salmonella* isolates, respectively. Use of these marbofloxacin-supplemented growth media mainly served as a selection step to reduce the number of isolates potentially susceptible to this antimicrobial, and thus limit the number of isolates to be tested by minimum inhibitory concentration (MIC) for resistance confirmation to marbofloxacin. MICs of marbofloxacin were determined for isolates grown on the marbofloxacin-supplemented media as well as for a limit of 100 selected generic *E. coli* isolates using the broth microdilution method in duplicate, and MICs results were interpreted as previously described [27]. To select the 100 generic *E. coli* isolates while ensuring their representativeness of both control and treated groups along the experiment, 3 isolates from different pigs of the control group, per sampling time, and 7 isolates from different pigs of the treated group, per sampling time, were used to characterize the evolution of MIC to marbofloxacin within the generic population of *E. coli*. In the current study, isolates with marbofloxacin MIC ≥ 4 µg/mL were defined as resistant (R), those with MIC = 2 µg/mL as intermediate (I) and the ones with MIC ≤ 1 µg/mL as susceptible (S) [27]. The disk diffusion method was carried out only for confirmed marbofloxacin-resistant isolates (MIC ≥ 4 µg/mL) to assess co-resistance to 14 clinically relevant antimicrobials, as used in the Canadian Integrated Program for Antimicrobial Resistance Surveillance (CIPARS) [33].

#### DNA extraction and genotypic susceptibility determination

Bacterial DNA extraction was performed on all isolates (*E. coli* or *Salmonella*) phenotypically resistant to marbofloxacin (MIC ≥ 4 µg/mL) as well as on the selected 100 generic *E. coli* isolates. DNA extraction was performed on these isolates using the Chelex solution (Bio-Rad Laboratories, Mississauga, Ontario, Canada). A solution of 10% Chelex was used to process 3 to 5 colonies from an overnight preculture on Tryptone Soya Agar (TSA) with 5% sheep blood at a temperature of 37 °C. The resulting suspension was vortexed for 10 s and placed in two subsequent dry baths, the first one being set at 55 °C and maintained for 30 min, followed by the second one at 98 °C and maintained for 15 min. Each sample was then centrifugated at 14,000 rpm for 5 min and the supernatant was kept at -20 °C for further analysis. Then, a Polymerase Chain Reaction (PCR) was carried out using the extracted DNA to identify mutations in quinolone resistance determining regions (QRDRs) of these strains. The PCR aimed at amplifying the target genes (*gyrA* and *parC*) in QRDRs to identify mutations in all strains phenotypically resistant to marbofloxacin using primers and conditions described previously [34]. The PCR products were sequenced using Sanger technique at the Génome Quebec platform (Montréal, Canada). In order to identify mutation on *gyrA* and *parC* genes, the obtained sequences were compared with those in the GenBank nucleotide database using the Basic Local Alignment Search Tool (BLAST) program available through the National Center for Biotechnology Information website as previously described [35].

### Statistical analysis

Bacterial counts of *E. coli* and *Enterobacteriaceae* were log_10_ transformed prior to data analysis to normalize distributions. All statistical analyses were performed with Student’s t-test using Excel 2019 software. The level of statistical significance was set at *p* < 0.05 for all analyses.

## Results

### General Animal condition and body weight

**Fig. 2:**
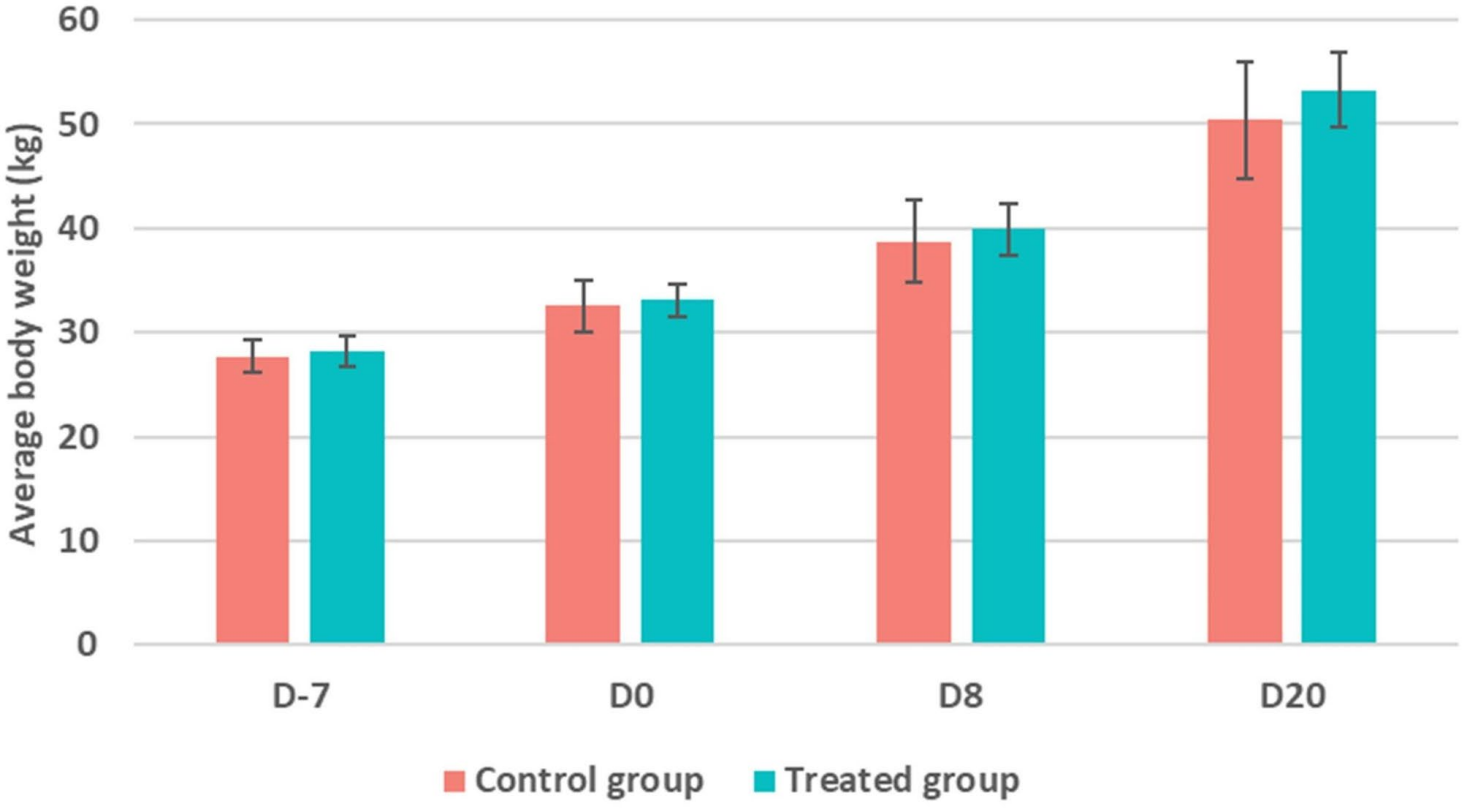
Distribution of body weight (kg) (mean ± standard deviation [SD]) of pigs in the control and treated groups. No significant difference was observed between the two groups for the 4 weighing times (*p* > 0.05). The intramuscular administration of marbofloxacin was carried out on D0

Clinically, none of the pigs in the 2 groups showed signs of diarrhea, anorexia or other signs of illness throughout the experiment. Furthermore, no signs of swelling, nodules, abscesses, or induration were observed at the injection site in any of the animals throughout the experimental period. It should be noted that no significant difference was observed between the average body weight of pigs in the two groups for the 4 weighing times (*p* > 0.05) (Fig. 2).

### Bacterial enumeration

#### *Enterobacteriaceae* enumeration

**Fig. 3:**
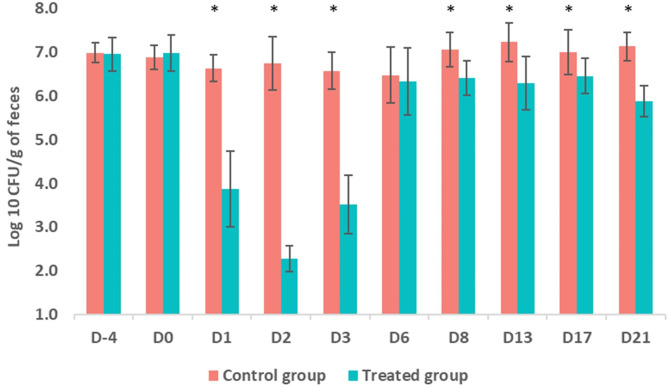
Distribution of the *Enterobacteriaceae* fecal population counts (mean ± standard deviation [SD]) in the control and treated groups throughout the experiment. The intramuscular administration of marbofloxacin was carried out on D0. * Indicates a statistically significant difference (*p* < 0.05)

During the acclimatization period (D-4) and prior to the IM administration of marbofloxacin or physiological solution (D0), the mean total of *Enterobacteriaceae* fecal population count was of 7 log_10_ CFU/g and 7 log_10_ CFU/g at D-4 and D0 in the treated group and 7.02 log_10_ CFU/g and 6.8 log_10_ CFU/g at D-4 and D0 in the control group (Fig. 3). There was no significant difference (*p* > 0.05) between the two groups for these two sampling times. However, after the IM administration of marbofloxacin (D1, D2, D3, D8, D13, D17 and D21), the mean total of *Enterobacteriaceae* fecal population count was significantly lower in the treated group compared to the control one (*p* < 0.05) (Fig. 3). It should be stressed here that the mean total of *Enterobacteriaceae* fecal population count on D2 was close to the detection limit (2 log_10_ CFU/g of feces) of our method. In addition, it was only on D6 following the IM administration of marbofloxacin that no statistically significant difference was observed between the treated and control groups in terms of *Enterobacteriaceae* fecal population counts. However, on D8, D13, D17, and D21 following the IM treatment with marbofloxacin, the total count of *Enterobacteriaceae* in the treated group consistently remained statistically lower than in the control group (*p* < 0.05). Moreover, although the *Enterobacteriaceae* fecal population counts in the treated group increased from D6 post-treatment, this increase did not return to the values observed at D-4 and D0 within the same group (*p* < 0.05) (Fig. 3).

### Generic *E. coli* population enumeration

**Fig. 4:**
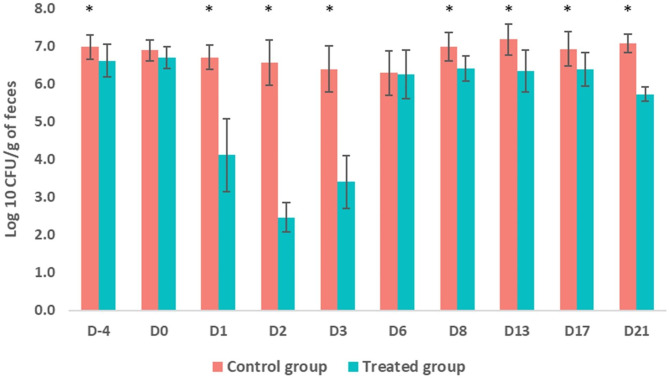
Distribution of the generic *E. coli* population counts (mean ± standard deviation [SD]) in the control and treated groups throughout the experiment. The intramuscular administration of marbofloxacin was carried out on D0. * Indicates a statistically significant difference (*p* < 0.05)

The enumeration profile of the fecal generic *E. coli* population follows that of the *Enterobacteriaceae* fecal population described above, with the only difference occurring at D-4, when a significant difference was observed between the treated and control groups (Fig. 4). Indeed, after the IM administration of marbofloxacin (D1, D2, D3, D8, D13, D17 and D21), except for D6, the mean generic *E. coli* population counts was significantly lower in the treated group compared to the control one (*p* < 0.05) (Fig. 4). Moreover, the mean generic *E. coli* population count on D2 was close to the detection limit (2 log_10_ CFU/g of feces) of our method (Fig. 4). On the other hand, a total of 569 generic *E. coli* isolates (obtained from both groups along the experiment on MacConkey agar plates not supplemented with marbofloxacin) were identified by MALDI-TOF MS analysis as *E. coli* and were frozen at -80 °C for subsequent analysis (characterization of phenotypic and genotypic AMR profile).

### *Salmonella* spp. detection

In the present study, no *Salmonella* isolates were detected in either group at any of the 10 sampling times along the study period.

### Antimicrobial susceptibility testing and sequencing results

In the present study, no isolates were identified by MALDI-TOF MS analysis as *E. coli* on MacConkey agar supplemented with marbofloxacin 1–4 µg/mL for either group of pigs. In the absence of marbofloxacin-resistant *E. coli* isolates (4 µg/mL), the evolution of marbofloxacin MIC was therefore characterized only on the 100 selected generic *E. coli* isolates (recovered from agar plates that had not been supplemented with marbofloxacin).

**Fig. 5:**
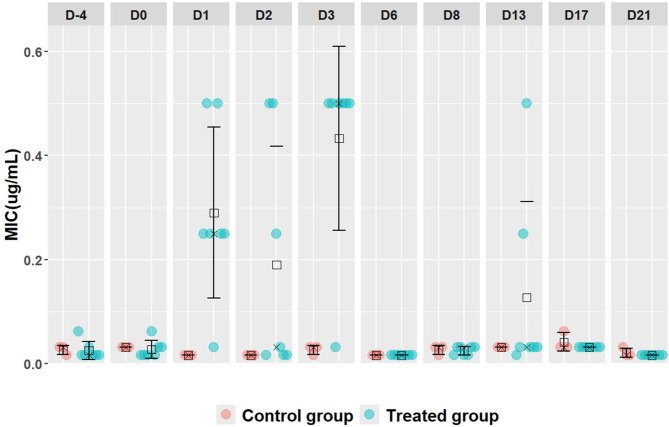
Distribution of MICs values for marbofloxacin in the generic *E. coli* population of both control and treated groups. For each distribution, mean and median values are shown by square and crosses, respectively. For each sampling time, 10 *E.coli* isolates (7 from the treated group and 3 from the control group) were characterized for MICs determination. The intramuscular administration of marbofloxacin was carried out on D0

For the MICs of marbofloxacin in this generic *E. coli* population, a significant increase in these values was observed at D1, D2 and D3 in the treated group compared with the control group (Fig. 5; Table 1). In fact, marbofloxacin MICs of up to 0.5 µg/mL were observed in some generic *E. coli* strains (*n* = 11) between D1 and D3 of the experiment, but these values were not observed beyond these 3 days (Fig. 5; Table 1).

**Table 1:** Evolution of mean MICs (and range) to marbofloxacin in the selected 100 generic *E. Coli* isolates from the treated and control groups of pigs

Groups/Time	D-4	D0	D1	D2	D3	D6	D8	D13	D17	D21
**Treated group**	0.02482	0.02702	0.2901	0.1899	0.433	0.016	0.0247	0.1273	0.0313	0.016
	(0.016–0.0625)	(0.016–0.0625	(0.0313-0.5)	(0.016-0.5)	(0.0313-0.5)	-0.016	(0.016–0.0313)	(0.016-0.5)	-0.0313	-0.016
**Control group**	0.0262	0.0313	0.016	0.016	0.0262	0.016	0.0262	0.0313	0.0417	0.0211
	(0.016–0.0313)	-0.0313	-0.016	-0.016	(0.016–0.0313)	-0.016	(0.016-0.0313)	-0.0313	(0.0131–0.0625)	(0.0313 − 0.016)
**Student test (** ***p*** **)**	0.4371	0.2674	0.0022*	0.0451*	0.0004*	1	0.4102	0.1084	0.2113	0.2113

* Statistically significant (*p* < 0.05)

In the absence of any identified *E. coli* strains phenotypically intermediate or resistant to marbofloxacin (MIC ≥ 1–4 µg/mL, respectively), we chose to sequence the *gyrA* and *parC* genes in all generic *E. coli* strains with MICs to marbofloxacin above 0.25 µg/mL (*n* = 17) (Table 1). However, sequencing results showed no mutations of those genes in the bacterial strains.

It is noteworthy that 119 and 6 bacterial isolates were isolated from MacConkey agar plates supplemented with marbofloxacin at 1 µg/mL and 4 µg/mL, respectively. After MALDI-TOF identification, all these isolates were identified as *Klebsiella pneumoniae*. In addition, some isolates on MacConkey agar that were not supplemented with marbofloxacin were also identified as *Klebsiella pneumoniae* by MALDI-TOF. A forthcoming study will focus into characterizing the phenotypic and genotypic resistance profiles of these isolates, while exploring how marbofloxacin affects the composition and diversity of the fecal microbiota of the sampled pigs, using the 16 S metagenomic sequencing method (Illumina MiSeq System) as previously described [20].

## Discussion

The present study represents the first investigation conducted in pigs to evaluate the effects of a single IM therapeutic dose of marbofloxacin on *E. coli* fecal load and the development of fluoroquinolone resistance in some *Enterobacteriaceae* bacteria (e.g., *E. coli* and *Salmonella*) derived from the fecal microbiota of these animals. Since marbofloxacin was not approved for swine use in Canada at the time of this project, the study was conducted under experimental conditions following the acquisition of an Experimental Studies Certificate (ESC) from the Veterinary Drug Directorate of Health Canada. The single dose of marbofloxacin used in this study (8 mg/kg (bw)) has been previously evaluated and showed efficacy in the treatment of acute porcine pleuropneumonia after experimental aerosol inoculation of pigs with *Actinobacillus pleuropneumoniae* [20], as well as in the treatment of clinical signs of respiratory disease in pigs [30]. Moreover, this same single IM dose of marbofloxacin was also used to assess the pharmacokinetic parameters of this drug in pigs [36], and to determine the antibacterial effect of this antimicrobial on intestinal bacteria through a pharmacokinetic/pharmacodynamic (PK/PD) in vitro and in vivo study [37]. To the best of our knowledge, this study represents the first in vivo evaluation of the effects of a single IM dose of marbofloxacin treatment on *Enterobacteriaceae* and *E. coli* fecal loads, as well as on the development of *E. coli* resistance to this antimicrobial up to 21 days post-treatment in pigs. With regard to the evaluation of the effect of marbofloxacin on *E. coli* in pigs, Lei et al. reported, by using a PK/PD ex *vivo* model, that marbofloxacin administered by the oral route in pigs at a dose of 2 mg/kg could have an effective bactericidal effect against *E. coli* [38]. The present study focused on clinically healthy animals, and the targeted age of the sampled pigs (60 days old) was motivated by the selection of pigs at times when they were not likely to be affected by respiratory and/or digestive infections. The single IM therapeutic dose of marbofloxacin in the present study was associated with a significant and rapid decrease in the fecal shedding of *Enterobacteriaceae* and *E. coli* populations. In fact, a 3-log, 4.5-log and 3.5-log reduction in the fecal shedding of both bacterial populations was observed after one, two and three days, respectively, following the IM administration of marbofloxacin, with the greatest impact in terms of this reduction, being observed on the second day after the start of treatment. These findings are similar to those of Lhermie et al. who reported that an IM administration of a single dose of 2 or 10 mg/kg of marbofloxacin to young bulls or veal calves significantly reduced fecal shedding of the *Enterobacteriaceae* population, with the maximum effect being observed on the 3rd day after the IM administration of this antimicrobial [39]. To discuss our results on the in vivo effect of the single IM administration of marbofloxacin in pigs on *E. coli* fecal shedding, we searched PubMed in January 2024, with the terms “marbofloxacin in pigs” or “marbofloxacin in swine” and “*E. coli*”, and “fecal shedding”. This same bibliographical search was updated again in June 2024. Surprisingly, no studies related to these topics were found, underscoring the novelty of our study. It is worth noting that the reduction in *E. coli* fecal shedding after the IM administration of marbofloxacin in the current study is closely comparable to the reduction observed after an oral administration of colistin sulfate (a cationic antimicrobial peptide) at the therapeutic dose of 50,000 IU/Kg for 5 days in an experimental model of Enterotoxigenic *E. coli* (ETEC: F4) infection in pigs [40]. These findings support the choice of marbofloxacin in some countries for the treatment of post-weaning diarrhea (PWD) in pigs [41–44]. The effectiveness of parenterally administered marbofloxacin in reducing *Enterobacteriaceae* and *E. coli* populations in pig intestines is mainly due to its PK, as the elimination of this antimicrobial in its active form toward intestines is prominent in pigs [45]. In fact, it was estimated that exposure to marbofloxacin in the small intestine segments of pigs was 2- to 8-fold higher than in plasma following a single IM administration of 8 mg/kg marbofloxacin [37]. This finding could also explain the persistent marbofloxacin effect on the fecal *Enterobacteriaceae* and *E. coli* populations in the treated pigs in the current study.

In the present study, no *Salmonella* isolates were identified in the feces of pigs from either group for the 10 sampling times. Data from the FoodNet Canada report in 2018 indicated a *Salmonella* prevalence of 19% in swine farms across the country [46], while other previous baseline studies conducted in Ontario, Alberta and Québec detected a prevalence ranging between 6% and 14% in the fecal samples from pigs [47–49]. This difference may be attributed to factors such as the small sample size (*n* = 20) and the individual sampling method used in our study, along with other factors related to pig breeding management. Although pigs are major sources of certain *Salmonella* serotypes linked to human salmonellosis [50], infected animals typically remain asymptomatic carriers, and *Salmonella* shedding from these animals is generally intermittent [51]. Within this context, a study by Arnold et Cook based on Bayesian methods showed that pooled fecal sampling is highly efficient compared to individual sampling [52]. It has also been shown that various factors including feeding practices, biosecurity measures as well as the general health status of pigs can also influence *Salmonella* prevalence in animals [53]. These factors likely contribute to the results observed in our study regarding the fecal absence of *Salmonella.* It is noteworthy to mention that nowadays, numerous biosecurity measures are applied by the Canadian swine industry throughout the PigSAFE program from the Canadian Pork Excellence (CPE) national platform to guarantee food safety and biosecurity of pig farms, which have certainly contributed to reducing the prevalence of *Salmonella* on farms [54].

Although marbofloxacin is a third-generation fluoroquinolone listed as a highest priority critically important antimicrobial in human medicine [55], the results of our study indicated that a single IM administration of marbofloxacin resulted in a slight increase in the MIC of this antimicrobial in the generic *E. coli* strains. However, this increase, observed only between D1 and D3 of the experiment, remained below the breakpoint of marbofloxacin resistance (4 µg/mL) and was not associated with the acquisition of mutations in *gyrA* or *parC* genes. One possible explanation to this transient increase in the MIC values could be due to an overexpression of the acrAB-TolC efflux pump in the resistant *E.coli* isolates, as previously reported by Bhardwaj et al. in an in vivo study in goats [56]. To the best of our knowledge, no studies have investigated the impact of a single IM administration of marbofloxacin on fluoroquinolone resistance within the fecal microbiota of pigs. However, it is worth noting that the pattern observed in the increase of the MIC of the generic *E.* coli strains in the current study is similar to the findings of Bhardwaj et al., who reported that the IM administration of 2 mg/kg of marbofloxacin to goats for 5 days led to a several-fold increase in the MIC of marbofloxacin (8 to 32 µg/mL) in the fecal *E. coli* populations during the treatment period (days 4 to 6) [56]. This was followed by a decrease in the MIC values to below 1 µg/mL starting on the 9th day after treatment [56]. Additionally, data from an European monitoring program on marbofloxacin susceptibility in bacteria isolated from diseased pigs revealed resistance rates of 7 to 10.4% among *E. coli* isolates, with no *Salmonella* resistant strains [27]. Importantly, these rates have shown no significant increase over the years, indicating a high susceptibility of these bacteria to marbofloxacin [27]. On the other hand, Huang et al., reported that an oral in-feed treatment of 90-day-old pigs with 5 mg/kg body weight of ciprofloxacin, a second-generation fluoroquinolone, administered daily for 30 days, led to an increase in the MIC of *E. coli* isolates for ciprofloxacin, reaching 128 µg/mL compared to the initial MIC of 1 µg/mL. While, after discontinuation of ciprofloxacin for 26 days, the MIC returned to its initial value of 1 µg/mL [57]. In contrast to our study, the prolonged daily oral administration of fluoroquinolones (e.g., over 30 days) could promote the selection of fluoroquinolone-resistant bacteria in the pig’s gut microbiota. However, it would be valuable for future research to examine the impact of repeated marbofloxacin treatments in the same animal on the development of fluoroquinolone resistance. Based on the results observed in the present study, we concluded that a single IM administration of marbofloxacin significantly reduced *E. coli* fecal charge while limiting the selection of marbofloxacin-resistant *E. coli* in pigs. These promising results need to be validated in a pig model of *E. coli* infection using a larger number of animals, while ensuring that this antimicrobial is administered only after confirming the bacterial strain’s sensitivity through antimicrobial susceptibility testing (e.g., disk diffusion, broth dilution).

## Conclusions

The results of the present study demonstrated that a single IM administration of marbofloxacin in healthy pigs significantly reduced the fecal shedding of both *Enterobacteriaceae* and total *E. coli* population over the three days following administration. On the other hand, no *Salmonella* isolates were recovered from any of the animals in both groups, underscoring the efforts of the Canadian pig industry to mitigate this microorganism on pig farms. Moreover, no *E. coli* strains were confirmed to be phenotypically resistant to marbofloxacin before or after treatment. Although there was a transient increase in marbofloxacin MICs post-treatment, it did not exceed 0.5 µg/mL in the generic *E. coli* strains, which remained below the marbofloxacin resistance breakpoint (4 µg/mL). These findings require validation in clinically sick pigs to support the targeted use of this antibiotic exclusively for treating limited number of clinical diseases, thereby reducing dependence on conventional metaphylactic group treatments in pigs.

## Data Availability

Data will be made available upon reasonable request to the corresponding author.

## Abbreviations

AMR: Antimicrobial resistance
AMU: Antimicrobial use
CIPARS: Canadian Integrated Program for Antimicrobial Resistance Surveillance
DNA: Deoxyribonucleic acid
EU: European Union
FAO: Food and Agriculture Organization
IM: Intramuscular
MALDI-TOF MS: Matrix-Assisted Laser Desorption Ionization-Time-of-Flight Mass Spectrometry
MIC: Minimum inhibitory concentration
QRDRs: Quinolone resistance determining regions
UNEP: United Nations Environment Programme
WHO: World Health Organization
WOAH: World Organisation for Animal Health
